# Fear of cancer recurrence in South Korean survivors of breast cancer who have received adjuvant endocrine therapy: a cross-sectional study

**DOI:** 10.3389/fpsyg.2023.1170077

**Published:** 2023-07-27

**Authors:** Seul Ki Park, Yul Ha Min

**Affiliations:** ^1^Department of Nursing, Daejeon University, Daejeon, Republic of Korea; ^2^College of Nursing, Kangwon National University, Kangwon-do, Republic of Korea

**Keywords:** breast cancer, fear of cancer recurrence, adjuvant endocrine therapy, risk factors, symptoms

## Abstract

**Introduction:**

Fear of cancer recurrence (FCR) is one of the most-prevalent psychological problems among cancer survivors, and younger females who have received endocrine therapy are particularly at risk of high FCR. The aim of this study was to determine the relationship between high FCR and factors related to it in South Korean patients with breast cancer who receive adjuvant endocrine therapy (AET).

**Methods:**

This cross-sectional study recruited 326 patients with breast cancer who had received AET. All participants were asked to complete a personal information sheet, the short form of the Fear of Progression Questionnaire, and the Menopause Rating Scale. The factors associated with high FCR were analyzed using association-rule analysis.

**Results:**

The mean FCR score was 32.24 (SD = 10.22), and 137 of the 326 (42.0%) patients had high scores (≥34). Hot flushes and sweating (moderate to extremely severe), depressed mood (moderate to extremely severe), irritability (moderate to extremely severe), invasive stage, taking tamoxifen, and being married were associated with high FCR.

**Conclusion:**

Since FCR was common in patients with breast cancer who received AET, patients at a greater risk of experiencing FCR must be screened and supported.

## 1. Introduction

Breast cancer is the most common cancer among females worldwide, with an incidence rate among South Korean females in 2020 of 21.1%, also making it the most common cancer in South Korea (2020 Trend Analysis of Cancer Incidence in Korea, 2023). In South Korea, the overall 5-year relative survival rate for patients with breast cancer increased from 79.2% during 1993–1995 to 93.8% during 2016–2020 (2020 Trend Analysis of Cancer Incidence in Korea, 2023). This increase was due to improvements in conventional treatments, including endocrine and targeted therapies, and to advances in diagnosis (Berry et al., 2005). Although adjuvant endocrine therapy (AET) has contributed significantly to reducing breast cancer recurrence and mortality rates, many survivors of breast cancer still suffer several physical and psychological problems attributable to the fear that cancer might progress or recur, such as hot flushes, sleep problems, joint pain, and depression (van Londen et al., 2014; Rosenberg et al., 2015; Sung et al., 2022). Such worries and fears can continue throughout the treatment period and also into survivorship (Dunn et al., 2015; Schapira et al., 2022).

Fear of cancer recurrence (FCR) is generally defined as the fear of the disease recurring or progressing in the same organ or a different area of the body (Vickberg and Johnson, 2003). Even though low FCR levels may promote self-monitoring behavior, which can lead to the detection of potential signs of relapse (Lee-Jones et al., 1997), high fear levels can impair daily functioning and lead to a reduced quality of life (Koch et al., 2014; Thewes et al., 2016; Ban et al., 2021; Tran et al., 2021). FCR is one of the most-prevalent psychological problems among cancer survivors across various tumor types. A systematic review of 130 studies found that 73% of cancer survivors reported some degree of FCR, 49% reported moderate to high degrees, and 7% reported a high degree (Simard et al., 2013). Moreover, several studies found that most breast cancer survivors had at least some FCR, even several years after being successfully treated (Koch et al., 2014; Nahm et al., 2021). In particular, breast cancer survivors have been found to suffer from higher levels or rates of FCR than other cancer survivors such as those of leukemia, and prostate, thyroid, and colorectal cancers (Koch-Gallenkamp et al., 2016; Vandraas et al., 2021; Luigjes-Huizer et al., 2022).

The potential risk factors for FCR in cancer survivors have been investigated in several studies. Demographic characteristics such as female sex (Luigjes-Huizer et al., 2022), lower age (Crist and Grunfeld, 2013; Simard et al., 2013; Koch-Gallenkamp et al., 2016; Guo et al., 2022; Luigjes-Huizer et al., 2022), lower education level (Koch-Gallenkamp et al., 2016; Guo et al., 2022), being unmarried (Guo et al., 2022), being employed (Janz et al., 2011), and having no religious belief (Niu et al., 2019) have been found to be related to a higher risk of FCR. Disease and treatment characteristics such as severe illness (Niu et al., 2019; Guo et al., 2022) and receiving chemotherapy (Luo et al., 2020), radiotherapy (Yang et al., 2017), or endocrine therapy (Götze et al., 2019) have been also identified as risk factors for FCR. Physical symptoms such as fatigue (Janz et al., 2011; Ellegaard et al., 2017), pain (Janz et al., 2011; Ellegaard et al., 2017), urinary symptoms (Gemmill et al., 2010), and discomfort due to treatment side effects (Nahm et al., 2021) were found to be associated with a higher risk of FCR. Psychological characteristics such as depression (Luo et al., 2020) and anxiety (Luo et al., 2020) were also significantly associated with FCR.

Meanwhile, a recent qualitative study that explored the experiences of breast cancer survivors related to FCR found that endocrine therapy and follow-ups were triggers of FCR, and that the survivors believed that their fear would disappear when the endocrine therapy and follow-ups ended (Şengün İnan and Üstün, 2019). Another study involving adult survivors of long-term cancer also highlighted that younger females who received endocrine therapy had a particularly high risk of FCR (Götze et al., 2019). Considering this, South Korean breast cancer survivors who receive AET could be at a high risk of FCR because they are diagnosed at an early age and their risk peaks at 40–50 years old, which differed from the characteristics of breast cancer survivors from other countries (Jung et al., 2022). Furthermore, they frequently suffer from menopause-related symptoms such as hot flushes and sweating, heart discomfort, and sexual problems (Moon et al., 2017; Sung et al., 2022) due to AET, sensations that are often interpreted as potential signs of recurrence or as late treatment effects (Heathcote et al., 2019).

It is therefore meaningful to explore FCR and factors associated with a higher risk of it among South Korean breast cancer survivors who received AET. However, an extensive review of the related literature did not identify any studies that have investigated the FCR and its related factors in South Korean patients with breast cancer who received AET. Previous studies that identified the risk factors for FCR in patients with breast cancer also only investigated those factors individually and did not explore possible combinations that could provide a comprehensive viewpoint of groups at a high risk of FCR.

In this study we therefore targeted South Korean breast cancer survivors who received AET, and aimed to determine the relationship between high FCR and the factors associated with it using association-rule analysis.

## 2. Materials and methods

### 2.1. Design and participants

This cross-sectional study involved 326 South Korean breast cancer survivors who received AET. Patients were recruited from the Asan Medical Center, a university-affiliated hospital in South Korea. At the time of admission to surgery, 1,447 consecutive females who had been histologically confirmed as having breast cancer were screened for eligibility from November 2016 to January 2018. The inclusion criteria were as follows: hormone-receptor-positive breast cancer, age of ≥20 years at diagnosis, and definitive surgery followed by AET irrespective of chemotherapy status. Females with distant metastases at diagnosis (stage IV breast cancer), local or regional recurrent tumors, or a medical history of psychiatric or neurologic illness were excluded. Among 1,447 survivors, 510 did not meet the inclusion criteria (273 were not indicated as having received AET, 153 had recurrent breast cancer, and 84 due to other reasons) and 574 did not consent to participate in this study (144 were not contacted and 430 declined to participate); 363 patients therefore participated in this study.

Patients completed a self-reported paper survey that included FCR at a visit to the clinic 3 or 6 months after AET initiation. Patients who had difficulty with the questionnaire could ask help from the assistant researcher. Data for 37 patients were excluded due to missing values, and so data from 326 patients were finally included in the present analyses. Data were de-identified and stored in a password-protected computer and file.

### 2.2. Measures

#### 2.2.1. Demographic and clinical characteristics

Information about the demographic and clinical characteristics of the participants was collected through self-reported questionnaires and from their electronic medical records. The demographic data included age, education level, marital status, religious belief, and employment status. The clinical characteristics related to disease and treatment included cancer history, family cancer history, family breast cancer history, menopause status, cancer stage, and treatment (type of surgery, chemotherapy, radiotherapy, and endocrine therapy agent).

#### 2.2.2. Measures of FCR

FCR was measured using the short form of the Fear of Progression Questionnaire (FoP-Q-SF) translated in Korean, a self-reported instrument that assesses fear of recurrence in patients. The scale showed adequate reliability (Cronbach’s α = 0.87) and validity in a sample of breast cancer patients (Mehnert et al., 2006). The FoP-Q-SF consists of 12 items regarding concerns related to disease progression, such as affective reactions, partner/family, work, and loss of autonomy and is scored on a five-point Likert scale ranging from 1 (‘never’) to 5 (‘very often’), with higher values indicating higher FCR levels. High FCR was defined as a total score on the FoP-Q-SF of 34 or higher (Sarkar et al., 2014).

#### 2.2.3. Measures of symptoms

Symptoms were examined using the Menopause Rating Scale (MRS) translated in Korean. The MRS is an internationally accepted tool for the evaluation of types and severity of menopausal symptoms and demonstrated a high level of internal consistency (Cronbach’s α = 0.87) with good validity (Heinemann et al., 2003). MRS is a self-reported scale consisting of 11 questions in three subscales: psychological, somatic, and urogenital. Symptom severity is rated on a five-point scale (0 = ‘no complaints’ to 4 = ‘very severe symptoms’). We classified those symptoms into two groups: physical and psychological. The physical symptoms were hot flushes and sweating, heart discomfort, sleeping problems, sexual problems, bladder problems, dryness of the vagina, and joint and muscular discomfort. The psychological symptoms were depressive mood, irritability, anxiety, and physical and mental exhaustion. We classified symptom severity into no (score = 0 points), mild (1 point), and moderate to extremely severe (2–4 points) symptoms.

### 2.3. Statistical analysis

Statistical analyses were performed using SPSS software (version 26.0, IBM, Armonk, NY) and R software (version 4.2.2). Descriptive statistics were used to describe all the demographic and clinical characteristics, symptoms, and FCR of the study sample. Univariate analyses of FCR were conducted using independent t-tests and ANOVA. Comprehensive characteristics of the high-FCR group were evaluated using association rules by applying the Apriori algorithm to determine factors associated with high FCR. Three main measures of the association-rule analysis were support, confidence, and lift. Support indicated the proportion of patients with high FCR and factors associated with FCR and in all patients. Confidence indicated the proportion of patients with high FCR among the patients with the factors associated with FCR. Lift indicated the ratio of experiencing high FCR in patients with the factors associated with FCR to experiencing high FCR in all patients. Rules with lift values >1 indicated a positive correlation, whereas rules with lift values <1 indicated a negative correlation. Lift values close to 1 indicated no association between high FCR and the factors associated with FCR.

## 3. Results

### 3.1. Demographic and clinical characteristics of the participants

This study included 326 patients diagnosed with breast cancer who were receiving AET. Their demographic and clinical characteristics are listed in Table 1. The participants were 22–70 years old, with a mean age of 46.94 years (SD = 8.36 years). Approximately 60% of them had at least a bachelor’s degree and 86.2% were married. About 50% were employed and 54.9% had a religious belief. A history of cancer, a family history of any cancer, and a family history of breast cancer were present in 4.0, 42.6, and 10.1%, respectively. About 70% were premenopausal. Most patients had stage I, II, or III cancer (87.1%) and had received conservative surgery (80.1%). Regarding previous breast cancer treatments, 34.0 and 85.0% had received chemotherapy and radiotherapy, respectively. Tamoxifen and aromatase inhibitor were being taken as hormone therapy agents by 79.8 and 20.2% of the patients, respectively.

**Table 1:** Demographic and clinical characteristics of participants and the univariate analysis for fear of cancer recurrence (*n* = 326).

Characteristics	M ± SD or n (%)	FCR (M ± SD)	*t*	*p*
Total		32.24 ± 10.22		
High FCR (≥34)	137(42.0%)			
**Demographic characteristics**				
Age	46.94 ± 8.36			
<50	218(66.9)	32.16 ± 10.08	−0.201	0.841
≥50	108(33.1)	32.40 ± 10.55		
Education level				
High school or below	128(39.3)	33.30 ± 10.92	1.466	0.144
College or above	198(60.7)	31.56 ± 9.71		
Marital status				
Married	281(86.2)	32.27 ± 10.38	0.273	0.785
Others (unmarried/separated/widowed/unknown)	45(13.8)	31.81 ± 9.34		
Religious belief				
Yes	179(54.9)	31.85 ± 10.10	−0.758	0.449
No	147(45.1)	32.71 ± 10.38		
Employment status				
Employed	166(50.9)	32.25 ± 10.08	0.011	0.991
Unemployed	160(49.1)	32.23 ± 10.40		
**Clinical characteristics**				
Cancer history				
Yes	13(4.0)	32.31 ± 12.39	0.024	0.981
No	313(96.0)	32.24 ± 10.14		
Family cancer history				
Yes	139(42.6)	33.53 ± 10.72	1.965	0.050
No	187(57.4)	31.29 ± 9.75		
Family breast cancer history				
Yes	33(10.1)	34.24 ± 12.96	0.957	0.345
No	293(89.9)	32.02 ± 9.87		
Menopause status				
Yes	93(28.5)	31.36 ± 10.70	−0.983	0.326
No	233(71.5)	32.59 ± 10.02		
Cancer stage				
*In situ*	42(12.9)	32.38 ± 9.45	0.095	0.924
Invasive (Stage I, II, or III)	284(87.1)	32.22 ± 10.34		
Type of surgery				
Mastectomy	65(19.9)	33.89 ± 10.69	1.459	0.146
Conservation	261(80.1)	31.83 ± 10.08		
Chemotherapy				
Yes	111(34.0)	31.99 ± 10.17	−0.317	0.752
No	215(66.0)	32.37 ± 10.27		
Radiotherapy				
Yes	277(85.0)	32.02 ± 10.07	−0.943	0.346
No	49(15.0)	33.51 ± 11.03		
Endocrine therapy agent				
Tamoxifen	260(79.8)	32.27 ± 10.05	0.086	0.931
Aromatase inhibitor	66(20.2)	32.14 ± 10.94		

M, Mean; SD, Standard Deviation; FCR, Fear of Cancer Recurrence.

The mean FCR score was 32.24 (SD = 10.22). The 326 patients included 137 (42.0%) with high FCR (≥34). None of the factors were significantly associated with FCR in the univariate analysis.

### 3.2. Physical and psychological symptoms of the participants

Table 2 lists the physical and psychological symptoms of the patients. Regarding physical symptoms, 78.8, 81.6, and 76.4% of the patients reported experiencing mild to extremely severe hot flushes and sweating, sleeping problems, and joint and muscular discomfort, respectively. Regarding psychological symptoms, 66.3 and 64.1% of the patients reported experiencing mild to extremely severe depressive mood and irritability, respectively, and 85.6% reported experiencing mild to extremely severe physical and mental exhaustion. All symptoms were found to be significantly associated with FCR in the univariate analysis. Patients who experienced more-severe physical or psychological symptoms tended to report higher FCR.

**Table 2:** Physical and psychological symptoms of the participants and the univariate analysis for fear of cancer recurrence (n = 326).

Symptoms	n(%)	FCR (M ± SD)	*F*	*p*
**Physical symptoms**				
Hot flushes and sweating				
No	69(21.2)	28.17 ± 10.58	12.419	<0.001
Mild	180(55.2)	32.06 ± 9.43		
Moderate to extremely severe	77(23.6)	36.31 ± 10.25		
Heart discomfort				
No	172(52.8)	29.59 ± 9.81	16.919	<0.001
Mild	143(43.9)	34.63 ± 9.47		
Moderate to extremely severe	11(3.3)	42.64 ± 12.40		
Sleeping problems				
No	60(18.4)	28.13 ± 9.93	16.119	<0.001
Mild	176(54.0)	31.29 ± 9.09		
Moderate to extremely severe	90(27.6)	36.84 ± 10.91		
Sexual problems				
No	141(43.2)	28.90 ± 9.88	16.000	<0.001
Mild	144(44.2)	34.12 ± 9.53		
Moderate to extremely severe	41(12.6)	37.12 ± 10.27		
Bladder problems				
No	171(52.5)	30.42 ± 9.72	6.818	0.001
Mild	123(37.7)	33.71 ± 10.35		
Moderate to extremely severe	32(9.8)	36.34 ± 10.55		
Dryness of vagina				
No	145(44.5)	30.12 ± 9.59	10.378	<0.001
Mild	152(46.6)	32.98 ± 9.97		
Moderate to extremely severe	29(8.9)	38.97 ± 11.40		
Joint and muscular discomfort				
No	77(23.6)	27.78 ± 9.93	11.592	<0.001
Mild	180(55.2)	32.98 ± 9.61		
Moderate to extremely severe	69(21.2)	35.29 ± 10.60		
**Psychological symptoms**				
Depressive mood				
No	110(33.7)	26.86 ± 8.90	44.693	<0.001
Mild	192(58.9)	33.76 ± 9.13		
**Moderate to extremely severe**	24(7.4)	44.71 ± 9.40		
Irritability				
No	117(35.9)	26.58 ± 8.45	48.763	<0.001
Mild	186(57.1)	34.31 ± 9.40		
Moderate to extremely severe	23(7.0)	44.26 ± 8.09		
Anxiety				
No	141(43.2)	26.26 ± 8.24	76.588	<0.001
Mild	171(52.5)	35.85 ± 8.69		
Moderate to extremely severe	14(4.3)	48.39 ± 7.21		
Physical and mental exhaustion				
No	47(14.4)	25.30 ± 9.24	22.132	<0.001
Mild	224(68.7)	32.28 ± 9.63		
Moderate to extremely severe	55(16.9)	38.00 ± 9.86		

FCR, Fear of Cancer Recurrence.

### 3.3. Relationship between high FCR and the factors associated with FCR

The relationship between high FCR and the factors associated with it was assessed using association-rule analysis. The top-five association rules with the highest lift are listed in Table 3. “Moderate to extremely severe hot flushes and sweating” and “moderate to extremely severe depressed mood” were found to be strongly associated with high FCR (support, confidence, and lift levels of 0.163, 0.867, and 2.033, respectively). The support level of 0.163 indicated that the proportion of patients with “moderate to extremely severe hot flushes and sweating,” “moderate to extremely severe depressed mood,” and “high FCR” among all patients was 0.163. The confidence level of 0.867 indicated that the proportion of patients with high FCR among the patients with “moderate to extremely severe hot flushes and sweating” and “moderate to extremely severe depressed mood” was 0.867. The lift level of 2.033 indicated that the ratio of experiencing high FCR in patients with “moderate to extremely severe hot flushes and sweating” and “moderate to extremely severe depressed mood” to experiencing high FCR in all patients was 2.033. “Moderate to extremely severe irritability,” “invasive stage,” “married,” and “tamoxifen” were also strongly associated with high FCR, with a lift value of >1.79.

**Table 3:** Association rule with top five lift.

Rules	Support	Confidence	Lift
{Hot Flushes and_Sweating (moderate to extremely severe), Depressed mood (moderate to extremely severe)} = > {high FCR}	0.163	0.867	2.033
{Depressed mood (moderate to extremely severe), Irritability (moderate to extremely severe)} = > {high FCR}	0.150	0.842	1.975
{Depressed mood (moderate to extremely severe), Stage_invasive} = > {high FCR}	0.166	0.815	1.913
{Depressed mood (moderate to extremely severe), Married} = > {high FCR}	0.154	0.790	1.854
{Irritability (moderate to extremely severe), Tamoxifen, Stage_invasive} = > {high FCR}	0.154	0.766	1.796

## 4. Discussion

This study examined the experience of FCR in South Korean patients with breast cancer who received AET, and comprehensively explored the characteristics of the high-FCR group. The mean FCR score was 32.24, with nearly 40% of patients reported experiencing high FCR. This value is lower than that of Niu et al. (mean = 38.8) in a Chinese population (Niu et al., 2019) but higher than that of Koch et al. (mean = 28.9) in a German population (Koch et al., 2014); both of those studies used the same instruments. The scores might differ due to differences in sampling such as age, time since diagnosis, or education level. In particular, the present study only targeted patients with breast cancer who received AET, which differs from previous studies involving general breast cancer survivors. However, the FCR scores in this study, which were determined among relatively young patients with short durations since diagnosis, was higher than that in the study of Koch et al., which involved long-term breast cancer survivors with a mean age of 65 years. This was consistent with previous findings that lower age (Crist and Grunfeld, 2013; Simard et al., 2013; Koch-Gallenkamp et al., 2016; Guo et al., 2022; Luigjes-Huizer et al., 2022) and shorter duration since diagnosis (Götze et al., 2019; Schapira et al., 2022) were significantly associated with higher FCR.

Our study found that depressed mood and hot flushes and sweating were strongly associated with high FCR. We found that 78.8% of the patients experienced mild or worse hot flushes and sweating. Hot flushes and sweating are some of the most common side effects experienced by patients with breast cancer receiving AET, with the reported prevalence exceeding 70% (Moon et al., 2017; Brett et al., 2018; Chumdaeng et al., 2020; Sung et al., 2022). These side effects are thought to result from disturbances of the temperature-regulating mechanism in the hypothalamus, and are triggered by reduced estrogen levels (Archer et al., 2011). A previous study found that the prevalence of hot flushes and sweating remains stable (at around 80%) regardless of whether the patient is in their first or fifth year of treatment (Moon et al., 2017). Symptom severity also remains high up to the fourth year of treatment (Moon et al., 2017). A previous qualitative study found that these patients suffered from sweating, mostly at night, which negatively affected their sleep quality and results in increased daytime fatigue (Ahlstedt Karlsson et al., 2019). These physical sensations can often be perceived signs of cancer recurrence or progression (Heathcote et al., 2019). It is not clear why hot flushes and sweating were more closely associated with high FCR than were the other physical symptoms. Since most previous studies investigated the relationship between FCR and general physical symptoms such as pain and fatigue (Ellegaard et al., 2017), it was difficult to compare the results of previous studies with our findings on specific symptoms experienced due to AET. However, it is obvious that most patients who receive AET experience hot flushes and sweating continuously during long treatment periods, which is closely related to high FCR. Patients who perceived their symptoms to be well under control in a previous study reported that they were less worried (Janz et al., 2011). Additional attention on hot flushes and sweating management by healthcare providers should therefore be included in survivorship care plans and monitored at follow-up.

This study found that psychological symptoms were strongly associated with high FCR. Depressed mood was identified as a factor closely associated with high FCR, since it was included in four of the top-five association rules. Irritability, which is a common symptom of anxiety, was also associated with high FCR, since it was included in two of the top-five rules. Consistent with our study, several other studies have found depression and anxiety to be significantly associated with high FCR in cancer survivors (Koch et al., 2014; Yang et al., 2018; Luo et al., 2020; Tran et al., 2021). Conversely, higher FCR was also strongly associated with increased risks of depression and anxiety (Tran et al., 2021; Lucas et al., 2023). Moreover, there was a positive correlation between anxiety and FCR scores at all time points throughout the treatment period (Schapira et al., 2022). Psychosocial factors such as depression, distress, and anxiety were also found to play much greater roles than clinical factors in FCR (Koch et al., 2014; Yang et al., 2018). Our study found that the patients with coexisting of depression and irritability experienced high FCR levels. This finding can be explained by the theoretical Lee-Jones model of FCR, which suggests that FCR shares similarities with anxiety and depression symptoms (Lee-Jones et al., 1997). A previous study investigated network connectivity between FCR, anxiety, and depressive symptoms in breast cancer patients have found that FCR, anxiety, and depression are comorbid and believed to cause exacerbations in one another during treatment and recovery (Yang et al., 2022). Specifically, rumination such as maladaptive cognitive process may interact with FCR, depression and anxiety symptoms (Liu et al., 2018). Since patients with cancer and anxiety or depression tend to spend more time thinking about the risk of recurrence and exhibit worse psychosocial adjustment (Pedersen et al., 2012), it is necessary to provide effective psychological support for those with breast cancer.

Among the demographic factors, patients who were married with depressed mood reported high FCR. Previous studies have found that married (Koch et al., 2014) or younger patients with children (Mehnert et al., 2009) were more likely to experience high FCR. However, contradictory results have also been found, with single patients being more likely than married patients to report high FCR (Yang et al., 2018). These differences in findings among several studies might be explained by the differences in research samples. A possible explanation for the high FCR in married patients in the present study is that they had concerns about the future welfare of the children (Mehnert et al., 2009) as well as grief about the burden imposed on partners and other family members (Connell et al., 2006). In addition, since the mean age of patients in this study was approximately 47 years and their children were likely to be younger, the fears reflect grief related to potential loss of opportunities to engage in the upbringing and observe the milestones of their children (Connell et al., 2006).

Regarding clinical factors, the finding that taking tamoxifen and experiencing irritability were associated with high FCR could be related to their low age. Tamoxifen is mostly prescribed to premenopausal females, whereas aromatase inhibitors are only prescribed to postmenopausal females. A previous study found that patients with breast cancer who used tamoxifen reported more menopausal symptoms than did those not taking tamoxifen (Harris et al., 2002). The premenopausal young patients might have experienced menopausal symptoms that they had never experienced before taking AET (Park et al., 2021), and these symptoms can lead patients to fear cancer recurrence or progression.

Consistent with other studies (Lane et al., 2019; Tran et al., 2021), we found that advanced cancer stage and depressive mood were associated with higher FCR. Patients with these factors may be more likely to perceive symptoms and physical changes as greater threats to their health and well-being, and consequently experience higher FCR (Lane et al., 2019).

This study had some limitations that should be addressed. First, it only investigated patients with breast cancer at a tertiary care hospital in Seoul, South Korea. This made it difficult to identify a sufficiently representative sample, and so the results cannot be generalized to the entire South Korean population with breast cancer who received AET. Second, the cross-sectional design of this study means that its findings cannot be used to determine any causal association between relevant factors and high FCR. Longitudinal studies are needed to further identify the causal association and monitor the trajectory of FCR. Third, the survey results of this study were subjective, and there might have been recall bias among the respondents.

## 5. Conclusion

We investigated FCR in South Korean patients with breast cancer who received AET and explored the relationship between high FCR and factors related to it. This study found that more than 40% of the patients experienced high FCR, which suggests the need for screening and management of FCR in patients with breast cancer taking AET. Among physical symptoms, hot flushes and sweating were found to have a strong association with high FCR. We found that psychological factors such as depressed mood and irritability were also strongly associated with high FCR. Being married, taking tamoxifen, and invasive stage were also associated with high FCR. These results suggest that patients with these characteristics should in particular be monitored and provided with appropriate interventions to alleviate FCR.

## Data availability statement

The raw data supporting the conclusions of this article will be made available by the authors, without undue reservation.

## Ethics statement

The studies involving human participants were reviewed and approved by Institutional Review Board of the Asan Medical Center. The patients/participants provided their written informed consent to participate in this study.

## Author contributions

SKP and YHM: conceptualization, methodology, formal analysis and investigation, writing— review and editing, and final approval of the manuscript. SKP: writing—original draft preparation. YHM: funding acquisition. All authors contributed to the article and approved the submitted version.

## Funding

This study was supported by the National Research Foundation (NRF-2018R1A1A3A04076879).

## Conflict of interest

The authors declare that the research was conducted in the absence of any commercial or financial relationships that could be construed as a potential conflict of interest.

## Publisher’s note

All claims expressed in this article are solely those of the authors and do not necessarily represent those of their affiliated organizations, or those of the publisher, the editors and the reviewers. Any product that may be evaluated in this article, or claim that may be made by its manufacturer, is not guaranteed or endorsed by the publisher.
